# The Effect of Antimicrobial Peptide (PA-13) on *Escherichia coli* Carrying Antibiotic-Resistant Genes Isolated from Boar Semen

**DOI:** 10.3390/antibiotics13020138

**Published:** 2024-01-31

**Authors:** Krittika Keeratikunakorn, Ratchaneewan Aunpad, Natharin Ngamwongsatit, Kampon Kaeoket

**Affiliations:** 1 Department of Clinical Sciences and Public Health, Faculty of Veterinary Science, Mahidol University, 999 Phuttamonthon 4 Rd., Salaya, Phuttamonthon, Nakhon Pathom 73170, Thailand; krittika.ker@student.mahidol.edu (K.K.); natharin.nga@mahidol.edu (N.N.); 2 Graduate Program in Biomedical Sciences, Faculty of Allied Health Sciences, Thammasat University, Rangsit Campus, Klongluang, Pathum Thani 12120, Thailand; aunpad@gmail.com; 3 Laboratory of Bacteria, Veterinary Diagnostic Center, Faculty of Veterinary Science, Mahidol University, 999 Phuttamonthon 4 Rd., Salaya, Phuttamonthon, Nakhon Pathom 73170, Thailand

**Keywords:** antibiotic-resistant bacteria, antimicrobial peptides, *Escherichia coli*, pig

## Abstract

A major global public health concern is antimicrobial resistance (AMR). Antimicrobial peptides (AMPs) are a potentially appropriate replacement for conventional antibiotics. The purpose of this research was to investigate the potential of the antimicrobial peptide PA-13, a synthetic AMP with 13 amino acids, to inhibit *E. coli* isolated from boar semen expressing antibiotic-resistant genes, as well as to determine the mechanism of action of this antimicrobial peptide on the bacterial membrane. The effectiveness of the bacterial inhibitory activity of PA-13 was tested at different concentrations by two fold serial dilutions in the range 0.488–500 µg/mL using the MIC and MBC methods. The impact of PA-13 on the bacterial membrane was examined at different concentrations of 0×, 0.5×, 1×, 2× and 4× of MIC using DNA leakage assay and electron microscopy. The PA-13 antibacterial activity result exhibited the same MIC and MBC values at a concentration of 15.625 µg/mL. When comparing DNA leakage at different MIC values, the results revealed that the maximum amount of DNA concentration was found two and three hours after incubation. For the results of SEM and TEM, the bacterial membrane disruption of this *E. coli* was found in the PA-13-treated group when compared with the negative control. In conclusion, synthetic PA-13 with its antibacterial properties is an alternative antimicrobial peptide to antibiotics in the pig industry.

## 1. Introduction

Antimicrobial resistance (AMR) is one of the global public health problems that humanity is facing, as reported by the World Health Organization [1]. In 2019 approximately 2.8 million Americans have been infected with antibiotic-resistant bacteria, and 35,000 of them have died [2]. Antibiotic misuse and overuse in the human, animal, and food production industry sectors contributed to antimicrobial resistance [2]. Pathogenic *E. coli* are an important and common problem in pig farms, and antibiotics have been used to treat them [3,4,5]. A rectal swab sample of diarrheal piglets revealed 100% resistance of *E. coli* to amoxicillin, and 97.3% of these bacteria exhibited multidrug resistance (MDR) [3]. Antibiotic-resistant genes have also been identified, including those from *E. coli* collected from diarrheal piglets and boar semen (*int1*, *mcr-1* and *mcr-3*) [3,6]. It has been shown that among the bacteria isolated from sows with endometritis and those showing vaginal discharge (i.e., *E. coli*, *Staphylococcus* spp., and *Streptococcus* spp.), *E. coli* accounted for 33.3% of the clinical cases, and most of the isolates showed multidrug-resistant genes. [4,7,8]. Therefore, the more antibiotics are used in pig farms, the more rapidly *E. coli* and other bacteria develop drug resistance. There are numerous causes that can contribute to endometritis, such as contaminated boar semen from artificial insemination or poor sanitation during the process of semen collection and preparation [9]. Endometritis can affect a sow’s reproductive capacity, causing abortion and the delayed onset of oestrus, resulting in a small litter size [8,10].

Colistin resistance in bacteria can be facilitated by plasmid-borne genes known as mobile colistin-resistant (*mcr*) genes. As of today, there are ten variations of the *mcr* gene, numbered *mcr-1* to *mcr-10* [11]. Globally, food, human, and animal samples have all been found to include the *mcr* gene [11]. When other antibiotics are ineffective against a bacterial infection due to resistance, colistin is the last medication to be administered. Thus, the existence of the *mcr* gene has detrimental effect to public health around the world because of colistin is considered a last-line treatment option for severe human infections [11]. Research on alternative methods that are able to reduce antibiotic use in pig farms has been conducted, particularly in terms of reducing antibiotic supplementation in the boar semen extender. These techniques include physical approaches such as single-layer centrifugation to exclude bacteria [12], storing the semen dose at low temperature (5 °C) without adding antibiotics [13], antimicrobial peptides (AMPs) or short antimicrobial lipopeptides [14], and other chemicals, such as lysozyme and kojic acid [15,16]. Every technique possesses individual advantages as well as disadvantages concerning antibacterial efficacy and its impact on semen quality.

Antimicrobial peptides (AMPs) appear to be an acceptable substitute for using conventional antibiotics. Recently, the Antimicrobial Peptide Database (APD) received 3257 AMPs [17]. Proline-rich antimicrobial peptides (PrAMPs), tryptophan and arginine-rich antimicrobial peptides, histidine-rich antimicrobial peptides, and glycine-rich antimicrobial peptides are instances of antimicrobial peptides identified as antimicrobial agents and could possibly serve as a form of treatment for antibiotic-resistant bacteria [18,19]. According to some studies [20,21,22], a possible mode of action of antimicrobial peptides is direct and rapid binding to the outer bacterial cell wall, such as lipopolysaccharide (LPS) in Gram-negative bacteria or teichoic acid in Gram-positive bacteria due to the difference in charge between the membranes of animals and bacteria [21,22,23]. In addition, lipopolysaccharides or teichoic acid are present on the outermost surface of bacterial cells; the positive charge of AMPs strongly interacts with the negative charge there [20,24,25], but it has a weak interaction with the positively charged animal membrane. More importantly, the predominant feature of AMPs is their ability to kill bacteria without causing damage to the host cell [26]. Recently, the new helical antimicrobial peptide PA-13 was shown to have broad-spectrum antibacterial action, especially against *Pseudomonas aeruginosa* with MDR, and was not harmful to animal cells [27]. This particular AMP showed a compromised result in terms of inhibiting Gram-negative bacteria with MDR. According to studies at pig farms in tropical countries, bacteria in boar semen are resistant to multiple antibiotics, such as amoxicillin, gentamicin, and colistin, commonly used in pig farms and supplemented in the boar semen extender [6,15]. It has also been demonstrated that *E. coli* isolated from boar semen has a unique MDR pattern [6]. However, no study has been reported on the efficacy of this PA-13 for inhibiting *E. coli* with MDR isolated from boar semen [6]. Instead of using antibiotics, the antimicrobial peptide PA-13 may be an alternative choice to reduce or replace antibiotics used in the boar semen extender. Therefore, this study aimed to investigate the antimicrobial peptide properties of “PA-13”, including whether to inhibit *E. coli* with an antibiotic-resistant gene, and the mechanism of action at the membrane level of *E. coli*.

## 2. Results

### 2.1. Antimicrobial Peptide Physicochemical Determination

The physicochemical properties of PA-13 was presented in Table 1.

### 2.2. Minimum Inhibitory Concentration (MIC) and Minimum Bactericidal Concentration (MBC)

The MIC and MBC of PA-13 against *E. coli* ATCC 25922 and *E. coli* isolated from boar semen (*int1* and *mcr*-*3* positive) was 7.813 and 15.625 µg/mL, respectively (Table 2). 

**Figure 1:**
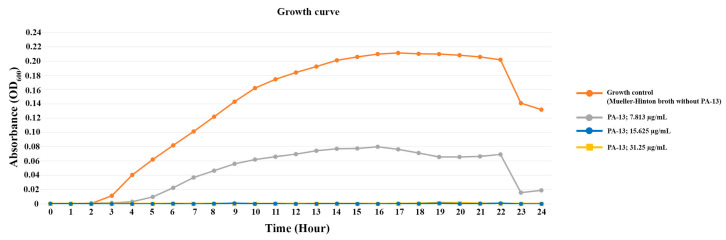
The growth curve of *E. coli* with antibiotic-resistant genes (*int1* and *mcr-3*) incubated with different concentrations of PA-13.

The growth curve of *E. coli* is shown in Figure 1. For the growth curve of the control (*E. coli* without PA-13) over 24 h, *E. coli* initiated the exponential phase and death phase at 2 and 22 h after incubation, respectively (Figure 1). PA-13 at a concentration 7.813 µg/mL can inhibit the growth of *E. coli*, as the exponential phase started 4 h after culture, and the bacterial concentration was less than the control. Moreover, the growth of *E. coli* with 15.625 and 31.25 µg/mL of PA-13 was not identified (OD_600_ = 0) during the 24 h of incubation. 

### 2.3. Leakage Assay

**Figure 2:**
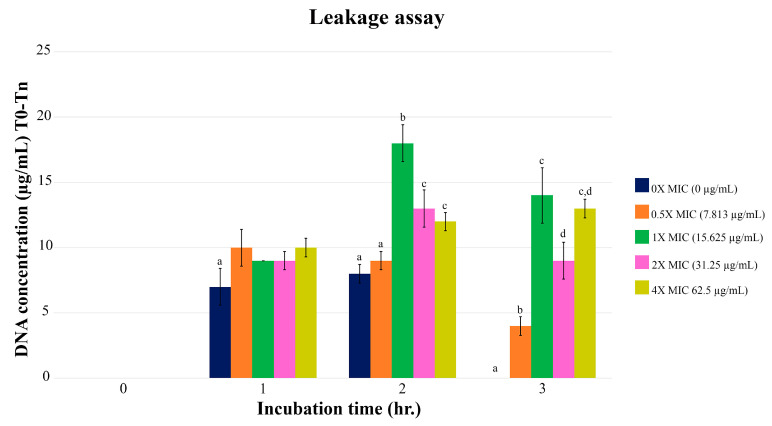
The DNA leak of *E. coli* after incubation with PA-13 at different concentrations, 0, 1, 2 and 3 h after incubation. ^a–d^ There was a significant difference between the leaked DNA from different concentrations of PA-13 (0×, 0.5×, 1×, 2×, and 4× of MIC) at the same incubation time (*p*-value < 0.05).

The leaking of *E. coli* DNA depended on the different concentrations of PA-13 (Figure 2). When comparing DNA leakage at different MIC values, the highest one was found two hours after the incubation. Comparing the leakage of DNA among different concentrations at 2 and 3 h of incubation, *E. coli* treated with PA-13 at a MIC value of 15.625 µg/mL showed the most significant DNA leak.

### 2.4. Scanning Electron Microscopy (SEM)

**Figure 3:**
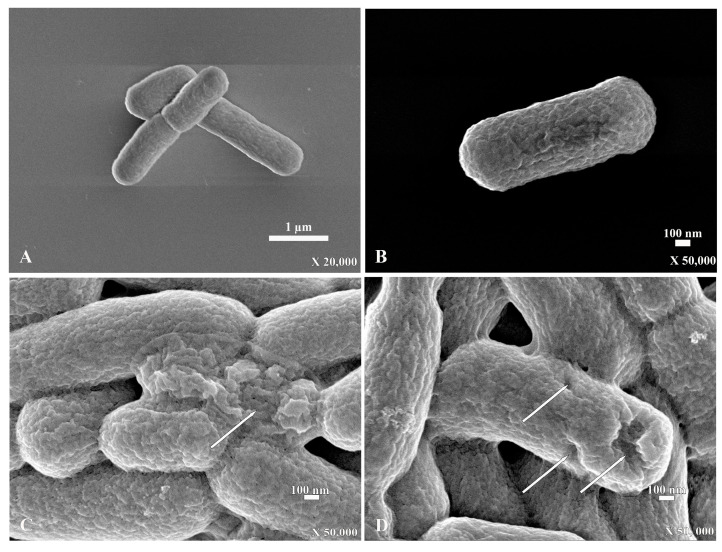
The scanning electron micrograph of *E. coli* with the normal shape of rod bacteria (**A**,**B**). The SEM of *E. coli* with a rupture on the surface (white arrow) after incubation with PA-13 at 15.625 µg/mL for 2 h at 37 °C (**C**,**D**).

For the scanning electron micrograph, *E. coli* that were incubated without PA-13 showed a normal morphology and rod shape with no traces of rupture on the bacterial surface (Figure 3A,B). However, *E. coli* incubated with PA-13 at 15.625 µg/mL for 2 h and 37 °C showed surface rupture (Figure 3C,D).

### 2.5. Transmission Electron Microscopy (TEM)

**Figure 4:**
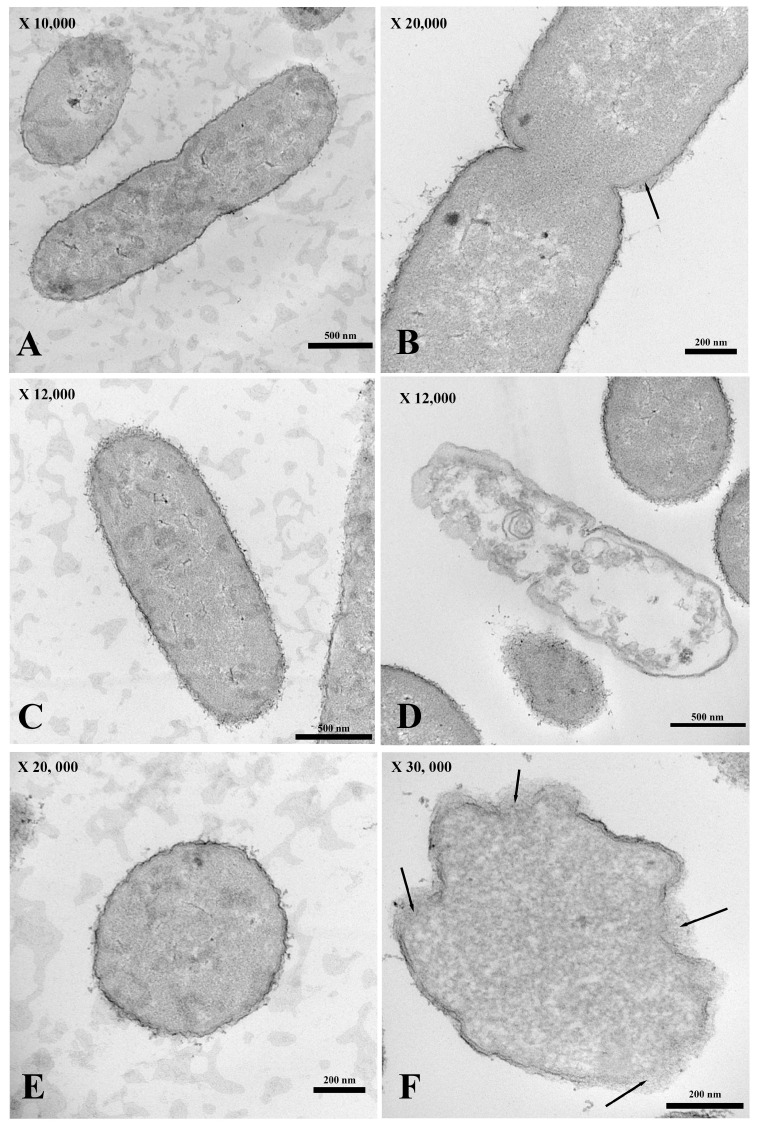
The longitudinal and cross-section of the transmission electron micrograph of *E. coli* both treated with PA 13 at 15.625 µg/mL (**B**,**D**,**F**) and untreated (**A**,**C**,**E**) for 2 h at 37 °C. *E. coli* were visibly ruptured (**B**,**F**; black arrow) and unable to maintain their rod shape (**D**,**F**), and there was a loss in the cytoplasmic content (**D**).

For the transmission electron micrograph, the membrane of *E. coli* incubated without PA-13 (Figure 4A,C,E) was not broken, as shown by its normal morphology (Figure 4A,C,E). There was no loss in the cytoplasmic content (Figure 4A,C,E), and the bacterial morphology remained capable of assuming the form of a rod (Figure 4A,C). However, *E. coli* treated with PA-13 at 15.625 µg/mL for 2 h at 37 °C (Figure 4B,D,F) resulted in an apparently torn membrane (Figure 4B,F), a loss of cytoplasmic content (Figure 4D), and an inability of the bacteria to maintain its rod shape (Figure 4D,F).

## 3. Discussion

It is documented that most of the bacteria contaminated in fresh boar semen are *E. coli* [1,2,3,6], which show resistance to amoxicillin and ceftriaxone, whereas *E. coli* isolated from pig rectal and nasal swabs are resistant to tetracyclines, penicillin, and chloramphenicol [6,28]. Since antibiotic-resistant genes can spread to the surrounding environments of pig farms and also to humans, *E. coli* that is resistant to antibiotics on a pig farm remains a risk factor to public health and pig production [29]. As demonstrated by a comparison of conventional and organic pig farms in Europe, completely reducing antibiotic use decreases the probability of antibiotic resistance [30]. According to a prior study, PA-13 is successful against multidrug-resistant (MDR) *Pseudomonas aeruginosa,* with the MIC values varying between 3.91 and 15.63 µg/mL, compared with gentamicin with a MIC value of more than 125 µg/mL [27]. Comparing with the present results, the MIC of PA-13 against antibiotic-resistant *E. coli* isolated from boar semen was found to be 15.625 µg/mL. This MIC value was comparable to the MIC for multidrug-resistant *Pseudomonas aeruginosa* isolated from a human case which was resistant to gentamicin [27].

The antimicrobial peptide is, therefore, a good option for replacing traditional antibiotics. In the present results, SEM and TEM clearly show that PA-13 causes damage to *E. coli* and a loss in the cytoplasmic content, which is similar to a study on *Pseudomonas aeruginosa* [27]. This is also in accordance with the mechanism of LI14 peptide, which has been effective against multidrug-resistant bacteria and inhibits the formation of bacterial biofilms [31]. The AMP has been reportedly used not only against aerobic bacteria but also against anaerobic bacteria. It has been demonstrated that the CM-A peptide can interact with the *Clostridioides difficile* membrane, resulting in damage to the bacterial cell, which is similar to the mechanism of PA-13 [32]. The results in the present study may be explained by earlier findings in that AMP has antimicrobial activity through direct and rapid binding to the outer membrane of bacteria, such as teichoic acid in Gram-positive bacteria and lipopolysaccharide (LPS) in Gram-negative bacteria [20,21,23]. In clarifying the mechanism by which AMP binds to bacteria, it has been elucidated that the presence of lipopolysaccharides or teichoic acid creates a positive charge with antimicrobial peptides and has a strong interaction with the negative charge on the outermost bacterial cell surface [20,21,24]. However, they have a weak interaction with the animal membrane, whose negative charge is located inside and is close to the cytoplasm [21,22]. This mechanism may at least, in part, explain the findings of *E. coli* membrane damage via SEM and TEM. Bacterial membrane damage may also account for the results of the bacterial DNA leakage observed in this study.

Considering these results together, antimicrobial peptides are dose-dependent, which has also been observed for the antibiotic agent. It is sufficiently documented that the bacterial cell is ruptured once the peptide concentration approaches an established threshold [33,34]. This is consistent with the present results in that the MIC concentration of PA-13 inhibited growth and increased the membrane rupture of *E. coli* to a greater extent than a lower MIC concentration (0.5× MIC). The differences between the charge in animal and bacterial cell membranes allow active AMP to attach to the bacterial membrane, subsequently causing membrane dysfunction, rupture (i.e., inducing membrane curvature and forming membrane pore), and lysis of the bacterial cell, which might explain the bacterial membrane rupture of *E. coli* found in this study [21,22,23,24]. Peptides damaged cell membranes at high concentrations, eventually causing them to break down, but at lower concentrations, they translocated to the cytoplasm where they interacted electrostatically with ribosomes or DNA [35,36,37]. However, it is worth noting that using antimicrobial peptides in sub-lethal concentrations may induce bacterial resistance to antimicrobial peptides [38]. In contrast, it has demonstrated that higher levels of the antimicrobial peptide, specifically pep + 7, do not enhance the ability to kill bacteria as effectively as lower levels due to the repulsion caused by the positive charge of nearby peptides [39]. This electrostatic repulsion leads to less attachment between the antimicrobial peptides and the bacterial membrane, resulting in a lower occurrence of bacterial cell rupture. This could partially account for the present DNA leakage assay outcome observed when a high concentration of PA-13 was examined. 

Antimicrobial peptides are a convincing alternative to traditional antibiotics, but their use is limited by a variety of factors, including their short half-life, high extraction costs, and instability against the protease [34]. For the short half-life of the antimicrobial peptides, the present results concerning the degree of bacterial rupture, indicated via DNA leakage, initially decreased after the incubation of PA-13 and *E. coli* for two hours, particularly at 0.5× MIC. This implies that antimicrobial peptides should be used with an optimal MIC in order to inhibit the growth of bacteria for a desirable period of time. In addition, alternative antimicrobial peptides should have a low MIC, high stability, and low toxicity [40]. Combining antimicrobial peptides with antibiotics is an additional method of resolving the limitations of these peptides. For example, the tetracycline and SAAP-18 peptide can be combined to inhibit *P. aeruginosa* [41,42]. However, further studies are needed to determine whether PA-13 can be used alone or in a combination with conventional antibiotics (i.e., gentamicin) in order to maintain boar semen qualities (i.e., motility, acrosome integrity and viability) [43] during the preparation of the boar semen extender for artificial insemination in the pig industry.

## 4. Materials and Methods

### 4.1. Bacterial Strains and Culture Conditions

*E. coli* previously isolated from boar semen and *E. coli* ATCC 25922 were kept in a culture collection at the Laboratory of Bacteria, Veterinary Diagnostic Center, Faculty of Veterinary Science, Mahidol University (Salaya, Phuttamonthon, Nakhon Pathom, Thai-land). All tested *E. coli* were cultivated in a brain heart infusion (BHI, Difco, Reno, NV, USA) medium and incubated at 37 °C for 16–18 h. The pre-culture was prepared via inoculating BHI broth with a single isolated colony and incubating at 37 °C for 16–18 h with shaking at 200 rpm. The pre-culture was added to the BHI broth at a concentration of 1% and grown at 37 °C prior to use. The PCR technique, which was described earlier by Keeratikunakorn et al. [6] was used to confirm the presence of antibiotic-resistant genes, and this technique has been reported by Nguyet et al. [3] to confirm the toxin genes.

### 4.2. Peptide Synthesis and Physicochemical Determination

The peptide in this study shown in Table 1 was synthesized, determined for physicochemical properties and validated for inhibiting *P. aeruginosa* by Klubthawee et al. [27]. In brief, PA-13 peptide was synthesized by solid-phase methods using 9-fuorenylmethoxycarbonyl (Fmoc) chemistry and purified by reversed-phase HPLC as trifuoroacetate salts (ChinaPeptides, Shanghai, China) [27].

### 4.3. Antimicrobial Activity

The inoculum of *E. coli* clinically isolated from boar semen (10^6^ CFU/mL) was tested with the PA-13 peptide at various concentrations. This MIC was determined using 96-well microtiter plates according to the Clinical and Laboratory Standards Institute (CLSI) guideline. The PA-13 peptide stock solution at 2 mg/mL was a twofold serial dilution and obtained concentrations of 500, 250, 125, 62.5, 31.25, 15.625, 7.8125, 3.906, 1.953, 0.976 and 0.488 µg/mL. One hundred microliters of the pre-culture were transferred into a 96-well plate, and one hundred microliters of PA-13 peptide at various concentrations were also added into the wells. A medium without PA-13 was conducted as the negative control. After 24 h of incubation, the optical density at 600 nm was measured using a microplate spectrophotometer (BMG LABTECH, SPECTROstar Nano, Ortenberg, Germany). The MIC values were recorded and defined as the lowest concentration of the PA-13 peptide that inhibited the growth of *E. coli* under the tested conditions. The MBC was defined as the lowest concentration of an antibacterial substance that killed ≥ 99.9% of the bacteria. After 24 h of incubation, the mixture of *E. coli* and the PA-13 peptide was used to inoculate BHI agar plates. The MBC of the peptide was investigated by observing the viability of the bacteria on the agar plate after incubation at 37 °C for 18–24 h. The experiments were performed in triplicate.

### 4.4. Bacterial Survival Assay

Bacterial survival after MIC testing was examined. After being transferred into a normal saline solution (0.85% NaCl), the *E. coli* in the culture media were properly mixed. A 0.5 McFarland standard (10^8^ CFU/mL) was used to measure the turbidity of the bacterial sample. Each well of the triplicate assays utilized 48-well plates containing 500 µL of bacterial suspension diluted in a Mueller–Hinton broth (Difco, Reno, NV, USA) to 10^6^ CFU/mL. This was combined with 500 μL of appropriate dilutions of PA-13 at the concentrations of 0× (growth control), 0.5×, 1×, and 2× of MIC. With the microplate spectrophotometer (BMG LABTECH, SPECTROstar Nano, Ortenberg, Germany), which was used to measure the OD_600_ values every hour for a 24 h period at 37 °C, a growth curve was constructed.

### 4.5. Leakage Assay

The *E. coli* was cultured in BHI broth (Difco, Reno, NV, USA) at 37 °C overnight. After culturing, the concentration of *E. coli* in the BHI broth (Difco, Reno, NV, USA) was adjusted by adjusting the OD_600_ into 1.5 by PBS of pH 7.2 (HiMedia, Thane, India). After that, PA-13 was added at different concentrations of 0×, 0.5×, 1×, 2×, and 4× of MIC and incubated at 37 °C. The DNA concentration was measured at 0, 1, 2, and 3 h after incubation. For the DNA concentration measurement, the sample was centrifuged at 8000 rpm for 2 min, and then the supernatant was used for the DNA concentration measurement using the protein analyzer (BioDrop, DKSH, Zurich, Switzerland) [44], and the sediment was further used for scanning electron microscopy and transmission electron microscopy [45].

### 4.6. Scanning Electron Microscopy (SEM)

The sediment from the leakage assay was used for scanning electron microscopy. The sediment was washed using PBS at pH 7.2 (HiMedia, Thane, India). After that, the samples were fixed by 2.5% glutaraldehyde (Electron Microscopy Sciences, UK) in PBS for 24 h. After the fixing process, the PBS was used for the washing process for 15 min and was repeated three times. For the next step, the samples were stained with 0.1% osmium tetroxide (Sigma-Aldrich, Burghausen, Germany) for 1 h, and washed using PBS at pH 7.2 (HiMedia, Thane, India). For the dehydration step, the sample was dehydrated by a graded series of ethanol at the concentrations of 70%, 80%, 90%, 95% and absolute ethanol. After processing, the samples were coated using platinum particles. Finally, the sample was observed under the field emission scanning electron microscope (JEOL, JSM-7610FPlus, Tokyo, Japan) [27].

### 4.7. Transmission Electron Microscopy (TEM)

The sediment from the leakage assay was used for transmission electron microscopy by classical conventional procedure. The sediment was washed using PBS at pH 7.2 (HiMedia, Thane, India) and fixed by using 2.5% glutaraldehyde (Electron Microscopy Sciences, London, UK) in PBS pH 7.2 for 24 h. Then, the samples were washed using PBS at pH 7.2 (HiMedia, Thane, India) and stained using 0.1% osmium tetroxide (Sigma-Aldrich, Germany) for 1 h, and washed again using PBS pH 7.2 (HiMedia, India). In the process of dehydration, the sample was dehydrated using ethanol at graded concentrations of 70%, 80%, 90% and 95%, as well as absolute ethanol. The samples were embedded in an araldite resin. The samples in araldite resin block were sectioned with a thickness of 60–90 nm and the section was stained with uranyl acetate and lead citrate. Finally, the sample was observed under the transmission electron microscope [27].

### 4.8. Statistical Analysis

The descriptive statistic was used for MIC and MBC. DNA concentration (T_0_ − T_n_) data were presented as the mean ± SD. A normal distribution test was performed using the Shapiro–Wilk test. In addition, the data analysis of parameters, including DNA concentration (T_0_ − T_n_) was performed using a one-way analysis of variance (ANOVA), and the means were compared among groups using Duncan’s test and the PASW Statistics for Windows, version 18.0 (SPSS Inc., Chicago, IL, USA). Statistical significance was determined as *p*-value < 0.05.

## 5. Conclusions

The present results reveal that *E. coli* isolated from boar semen is inhibited by PA-13 with a MIC of 15.625 µg/mL, which provides evidence that this antimicrobial peptide has the ability to damage bacterial membranes as shown via the DNA leakage assay and sperm morphology study with SEM and TEM. However, further study is needed to determine whether the PA-13 with its antimicrobial property could be an alternative to conventional antibiotics in the boar semen extender.

## Figures and Tables

**Table 1:** Physicochemical properties of the PA-13 peptide.

Peptide	Amino Acid Sequence	Number of Amino Acids	Molecular Weight (g/mol)	Net Charge	Hydrophobicity	Percentage of Hydrophobic Residues
PA-13	KIAKRIWKILRRR	13	1736.25	+7	0.678	46%

**Table 2:** MIC and MBC of *E. coli* using PA-13.

Sample	Antibiotic-Resistant Genes	Toxin Gene	MIC (µg/mL)	MBC (µg/mL)
*E. coli* ATCC 25922	-	-	7.813	7.813
*E. coli* from boar semen	*int1*, *mcr-3*	-	15.625	15.625

## Data Availability

Data is contained within the article.
